# Pandemic Influenza and Pregnant Women

**DOI:** 10.3201/eid1401.070667

**Published:** 2008-01

**Authors:** Sonja A. Rasmussen, Denise J. Jamieson, Joseph S. Bresee

**Affiliations:** * Centers for Disease Control and Prevention, Atlanta, Georgia, USA

**Keywords:** pregnancy, women, influenza, H5N1, avian influenza, pandemic influenza, antiviral medications, influenza vaccine, perspective

## Abstract

Planning for a future influenza pandemic should include considerations specific to pregnant women. First, pregnant women are at increased risk for influenza-associated illness and death. The effects on the fetus of maternal influenza infection, associated fever, and agents used for prophylaxis and treatment should be taken into account. Pregnant women might be reluctant to comply with public health recommendations during a pandemic because of concerns regarding effects of vaccines or medications on the fetus. Guidelines regarding nonpharmaceutical interventions (e.g., voluntary quarantine) also might present special challenges because of conflicting recommendations about routine prenatal care and delivery. Finally, healthcare facilities need to develop plans to minimize exposure of pregnant women to ill persons, while ensuring that women receive necessary care.

Influenza pandemics occur when a new influenza type A virus to which the population has no immunity emerges, spreads efficiently between humans, and results in worldwide outbreaks of severe disease. Pandemics occur infrequently but can be devastating in terms of the effects on illness and mortality. Most influenza experts consider influenza pandemics inevitable (*1*). The emergence of avian influenza A virus (H5N1) as a cause of severe human infections has increased concerns about an impending pandemic. Although human disease caused by influenza (H5N1) is a rare event (*2*), the virus has become endemic among bird populations in some areas of Asia and has continued to spread geographically and to broaden its host range. Concerns that the virus might acquire the ability to efficiently spread between humans have led public health authorities to accelerate preparations for pandemic influenza.

A key component of pandemic preparedness (*3*) involves addressing the specific needs of vulnerable populations, including pregnant women. Pregnant women are at high risk for severe complications of influenza during interpandemic periods (*4*) and previous pandemics (*5*–*8*). In addition, some studies suggest an increased risk for adverse outcomes among infants born to mothers infected with influenza during pregnancy (*9*–*12*). Special considerations for pregnant women should be addressed in all 3 categories of public health response to pandemic influenza—nonpharmaceutical interventions, antiviral medications, and vaccines. Many articles have discussed issues regarding pandemic influenza in the general population, but limited attention has been given to the effects on the pregnant woman and her fetus. This article focuses on issues regarding pregnant women that should be considered by public health and medical professionals as they prepare for a future influenza pandemic.

## Pandemic versus Seasonal Influenza

Influenza viruses that infect humans are classified into 3 principal types (A, B, and C), of which types A and B are important causes of human disease. Types A and B are associated with seasonal epidemics; only type A viruses have caused pandemics. Influenza A viruses are further classified on the basis of 2 surface proteins, hemagglutinin (H) and neuraminidase (N). Minor mutations that result in subtle changes in these proteins (antigenic drift) occur continuously. Because these mutations produce viruses that can be sufficiently different antigenically from previous influenza viruses, influenza vaccines must be updated annually. More dramatic changes in the surface proteins of influenza viruses, through mutation of nonhuman (e.g., avian or swine) viruses or reassortment of human and nonhuman viruses, result in the creation of novel human subtypes (termed antigenic shift). When novel subtypes that can be efficiently transmitted among humans emerge within a population that lacks immunity, an influenza pandemic can occur (*3*).

Avian species are an important reservoir for influenza virus, but avian influenza viruses do not typically infect humans. However, in 1997, human exposure to ill birds infected with the avian influenza A (H5N1) led to a severe outbreak in Hong Kong Special Administrative Region, People’s Republic of China.The H5N1 virus subtype reemerged in early 2003, and since that time, the virus has caused poultry and wild bird illnesses in >50 countries. In addition, 321 confirmed human cases of influenza (H5N1) in 12 countries have been reported to the World Health Organization, 194 of which have resulted in death (*2*). Thus far, influenza (H5N1) transmission has been predominantly bird-to-human, but the ongoing avian disease and occasional human disease have raised concerns about the possibility that this virus could gain the capacity for efficient human-to-human transmission and possibly lead to an influenza pandemic (*3*). Although influenza (H5N1) represents the greatest current threat for a pandemic virus, global public health authorities recommend increased vigilance for any novel influenza virus infections in humans as a cornerstone of pandemic preparedness.

## Effects of Influenza on Pregnant Women

Pregnancy has been a risk factor for increased illness and death for both pandemic and seasonal influenza. The increased risk is believed to be related to several physiologic changes that occur during pregnancy. Because of mechanical and hormonal alterations that occur during pregnancy, several changes also occur to the cardiovascular and respiratory systems, including increased heart rate, stroke volume, oxygen consumption, and decreased lung capacity (*13*). Relevant immunologic alterations also occur during pregnancy, with a shift away from cell-mediated immunity toward humoral immunity. This shift can render pregnant women more susceptible to, or more severely affected by, certain viral pathogens, including influenza (*14*).

Although appropriate nonpregnant control groups were generally not available, mortality rates among pregnant women in the pandemics of 1918 and 1957 appeared to be abnormally high (*5*,*7*). Among 1,350 reported cases of influenza among pregnant women during the pandemic of 1918, the proportion of deaths was reported to be 27% (*5*). Similarly, among a small case series of 86 pregnant women hospitalized in Chicago for influenza in 1918, 45% died (*6*). Among pregnancy-associated deaths in Minnesota during the 1957 pandemic, influenza was the leading cause of death, accounting for nearly 20% of deaths associated with pregnancy during the pandemic period; half of women of reproductive age who died were pregnant (*7*).

Pregnant women have also been shown to be at increased risk for influenza complications during interpandemic periods (*15*). In a large study of >4,300 women of reproductive age during 19 interpandemic influenza seasons, pregnant women were compared with postpartum women (a group considered to be most similar to pregnant women demographically and with regard to their health) and were found to be significantly more likely to be hospitalized for a cardiopulmonary event during the influenza season (*4*). The risk for hospitalization increased as pregnancy progressed, with women at term nearly 5 times more likely to be hospitalized than postpartum women (*4*). Similarly, during 3 influenza seasons in the late 1970s, rates of medical visits for acute respiratory disease were more than twice as high among pregnant women than nonpregnant women (*16*). At particularly high risk during the influenza season are pregnant women with underlying medical conditions for which influenza vaccination is recommended, such as asthma (*17*). On the basis of these data, pregnant women should be considered a population for which special considerations for prevention and treatment for influenza need to be made.

## Effects of Influenza on the Fetus

Although certain infections are well recognized to increase the risk for adverse pregnancy outcomes, the effects of maternal influenza infection on the fetus are not well understood. Viremia is believed to occur infrequently in influenza (*18*), and placental transmission of the virus also appears to be rare (*19*). However, even in the absence of fetal viral infection, animal studies suggest that adverse effects can still occur. Prenatal influenza infection in the mouse has been associated with histopathologic changes in the brain (*20*) and behavioral alterations (*21*) in offspring. Although influenza virus RNA has not been detected in the fetal brain, these changes suggest that fetal effects could be secondary to the maternal inflammatory response, rather than the result of a direct viral effect (*22*).

Adverse pregnancy outcomes have been reported following previous influenza pandemics. During the influenza pandemic of 1918, remarkably high rates of spontaneous abortion and preterm birth were reported (*5*,*6*), especially among women with pneumonia (for example, in 1 study, >50% of pregnancies in which the pregnant woman had influenza and accompanying pneumonia were not carried successfully to term) (*5*). During the Asian influenza pandemic of 1957, studies suggested a possible increase in defects of the central nervous system (*10*–*12*) and several other adverse outcomes, including birth defects, spontaneous pregnancy loss, fetal death, and preterm delivery (*8*). Studies of the effects of seasonal influenza infection on the fetus have been contradictory. A small increased risk for birth defects in general and for specific birth defects have been observed in some but not all studies (*9*). Using data from a recent case-control study, investigators showed that mothers of infants with any type of birth defect were slightly more likely to report influenza during early pregnancy than mothers of control infants (adjusted odds ratio 1.4; 95% confidence intervals 1.3–1.6), with statistically significant associations for cleft lip with or without cleft palate, and neural tube and congenital heart defects. Verification of maternal report of influenza illness with prospectively collected clinical data was possible for similar numbers of case and control infants (*9*), which suggests that recall bias was unlikely to explain the association. The risk associated with influenza was reduced for women who received treatment with antifever medications and for those who had taken folic acid before and during early pregnancy (*9*).

Associations between maternal influenza infection after both pandemic and seasonal influenza and outcomes observed long after birth have been reported. Associations between maternal influenza infection and childhood leukemia (*23*), schizophrenia (*24*), and Parkinson disease (*25*) have been suggested by some studies. Even if the influenza virus does not have a direct effect on the fetus, fever that often accompanies influenza infection could have adverse effects. Both animal and human epidemiologic studies suggest that hyperthermia is associated with an increased risk for adverse outcomes (*26*), especially neural tube defects (*27*). Factors that might attenuate this risk include shorter fever duration (*28*), use of fever-reducing medications (*28*–*30*), and use of folic acid–containing supplements (*29*,*31*).

More study is needed to better understand the fetal risks of maternal influenza infection. However, data from previous pandemics, although limited, suggest that pregnancy loss and preterm delivery could be important issues during a future influenza pandemic. Information on seasonal influenza indicates that influenza infection or its accompanying hyperthermia might also increase the risk for certain birth defects. Data on these potential risks to the fetus, combined with available information on risks for influenza infection on maternal health, provide ample support for considering pregnant women a high-risk population in an influenza pandemic.

## Pandemic Influenza Response for Pregnant Women

### Nonpharmaceutical Interventions

A main component of the public health response to pandemic influenza will be nonpharmaceutical interventions to mitigate disease rates and severity and the societal impact of the pandemic beyond health outcomes. The Centers for Disease Control and Prevention recently released guidance for nonpharmaceutical interventions during a pandemic that focuses on isolation of ill persons, voluntary quarantine of households with ill persons, and social distancing techniques (e.g., avoiding crowded settings, closing schools and child care centers) to limit exposure to ill persons (*32*). However, these recommendations present special challenges for pregnant women. For example, pregnant women will need guidance on how to protect themselves from becoming infected (e.g., use of protective devices) if they are quarantined with or directly providing care for ill persons. Responsibilities of pregnant women as members of the workforce and as caregivers of their children and other family members may further complicate their adherence to public health recommendations. In addition, because healthy pregnant women will continue to require both outpatient prenatal care and inpatient delivery services during a pandemic, they might be more likely to be exposed to clinical settings where ill persons are receiving care. Given the potential risk to women in clinical settings, guidance will need to be developed regarding whether some routine prenatal care visits could be omitted. Healthcare facilities need to develop plans to ensure that pregnant women receive necessary care, but with minimal exposure to ill persons or their contacts. In addition, plans for care and delivery of pregnant women with confirmed influenza or recent exposure must ensure that these women receive appropriate care without unduly exposing other healthy pregnant women and their infants to illness. An appropriate strategy to address these issues might include designating a location and staff for care of pregnant women and their newborns, separate from those used by patients with influenza. Another strategy could include developing an algorithm that triages pregnant patients on the basis of pregnancy stage and symptoms to ensure that pregnant women most in need of attention receive care, but avoid the risk of influenza exposure when that risk might be greater than the benefit of care. Consideration of these strategies as part of overall community pandemic planning activities will be essential.

Experience from the international outbreak of severe acute respiratory syndrome (SARS) (*33*) can shed light on how to approach these complicated issues. In Toronto, obstetric services were moved into a newly designed facility that had entrances, elevators, and air-handling systems that were separate from the rest of the hospital. Hospital staff, patients, and visitors were screened at the hospital entrance for SARS symptoms and to ensure that they had not visited a SARS-affected area. Staff members wore N95 respirator masks, face shields or eye protection, gowns, and non-latex gloves, and employed frequent hand washing with ethanol-based gels. Patients were limited to 1 visitor during labor and delivery, and no visitors were allowed on postpartum wards. All patients and visitors wore N95 respiratory masks. The length of postpartum stay was decreased and, after discharge, women were instructed to stay at home under quarantine for 10 days; a nurse visited them on their third day postpartum. Healthcare workers were asked to observe work quarantine; they were encouraged to go directly to work and home minimizing contact with the public (*34*). In Hong Kong, obstetric services were transferred to a hospital separate from hospitals in which SARS cases were managed. Women were discharged sooner after delivery, and all obstetric services considered nonessential (e.g., routine ultrasonography and prenatal diagnosis) were temporarily suspended (*33*).

### Antiviral Medication Use by Pregnant Women

During a pandemic, 2 pharmaceutical options—antiviral medications and vaccination—will be available to reduce the expected illness and death. Given that a vaccine is unlikely to be available for a substantial portion of the population at the beginning of a pandemic, antiviral medications are expected to play an important role in the response to pandemic influenza, both for postexposure prophylaxis and for influenza treatment. Two antiviral medications are currently recommended for treatment and prophylaxis of influenza in humans (*15*). These medications, both neuraminidase inhibitors, are available in oral (oseltamivir [Tamiflu]) and inhaled (zanamivir [Relenza]) forms, and make up the bulk of stockpiled anti-influenza medications. Two additional anti-influenza medications, the M2 ion channel blockers rimantidine and amantadine, are currently not recommended for use because of high rates of resistance among circulating human influenza A viruses and some avian influenza viruses.

As is the case with >90% of medications introduced in recent years (*35*), insufficient information on oseltamivir and zanamivir is available to assess potential risks to the fetus. This is reflected by their category C use-in-pregnancy rating from the US Food and Drug Administration (i.e., insufficient information available to assess their potential risk and benefit during pregnancy) (*35*). Animal studies have shown no evidence of increased risk for adverse effects for either medication (*36*,*37*), but animal data do not always predict the effects on human pregnancies (*35*). Human data are very limited. Among 61 pregnant women exposed to oseltamivir in the post-marketing period, most pregnancies had a normal outcome (*36*). Single cases of trisomy 21 and anencephaly were reported among these exposed pregnancies, but these cases were not believed to be causally related to oseltamivir exposure. Three pregnancies were inadvertently exposed to zanamivir during the clinical trials, with one ending in spontaneous abortion, one in elective termination, and one in an outcome with no apparent adverse effects (*37*). The bioavailability (the proportion of active drug that reaches the systemic circulation) of zanamivir is lower (12%–17%) than that of oseltamivir (≈80%) (*38*), leading some to suggest that it might be preferred during pregnancy (*3*).

Many pregnant women will require treatment with other medications, such as antibiotics for secondary bacterial pneumonia and antipyretic medications for fever control. Healthcare providers need access to information on these medications and their safe use during pregnancy so that effects on the fetus can be taken into account.

Another issue to consider is that even in the case of serious exposure or illness, pregnant women might fail to comply with recommendations for use of antiviral medications because of concern for the health of the fetus. This emphasizes the importance of communication of the risks and benefits of medications and the serious nature of untreated influenza. As with all communications related to pandemic influenza, messages must be culturally and linguistically appropriate and conducted at an appropriate level of literacy to ensure such efforts are inclusive, given the diversity of the population of pregnant women.

Further research to understand the effects of anti-influenza medications on the pregnant woman and her fetus is essential to guide treatment recommendations during a future pandemic. Although exposures to these medications are likely to be rare in the pre-pandemic period, collection of data on these exposures in a pregnancy registry, as has been used to collect data on other rare exposures (*35*), could provide important data to guide pandemic recommendations. In the absence of additional information, healthcare providers will need to consider the type of exposure, risk for serious illness or death, and trimester of pregnancy when weighing the risks and benefits of these medications to the woman and her fetus. Guidelines for the pandemic scenario, similar to those developed for management of sporadic avian influenza (H5N1) infection (*39*), could assist healthcare providers in weighing these risks and benefits.

### Use of Pandemic Influenza Vaccine among Pregnant Women

Once available, a vaccine will be a vital component of the public health response to pandemic influenza. Given their increased risk for illness and death during pandemic influenza, pregnant women should be considered a high priority for receipt of influenza vaccine. Several studies have demonstrated no adverse fetal effects when women received inactivated vaccine during pregnancy (*15*,*40*). Both the Advisory Committee on Immunization Practices and the American College of Obstetricians and Gynecologists recommend annual vaccination with trivalent inactivated influenza vaccine for women who will be pregnant during the influenza season (October–mid May) to prevent seasonal influenza (*15**,**40*). (Live, attenuated, influenza virus vaccine, available as an intranasal spray, is not approved for use during pregnancy, given the theoretical risk associated with use of live vaccine during pregnancy.) Inactivated influenza vaccine is recommended in all 3 trimesters of pregnancy (*15*,*40*). Despite these recommendations, compliance has been low (*15*), probably because of concerns among women and their healthcare providers regarding the safety of vaccination during pregnancy. Development of culturally and linguistically appropriate messages will be necessary to ensure that pregnant women receive information regarding care required for their health and that of their fetus in the event of a future pandemic. Professionals who develop these messages need to be aware that some women will have limited access to healthcare services. Innovative strategies will be needed to ensure that these messages reach them.

### Incorporating Issues of Pregnant Women into Preparedness Exercises

Pandemic influenza planning that specifically addresses the concerns of pregnant women is critical because special issues need to be considered for this high-risk group. Pandemic influenza preparedness exercises should include scenarios in which issues specific to pregnant women require attention. In the event of an influenza pandemic, identification and close monitoring of pregnant women will be important, given their increased risk for influenza-associated illness and death. Intake procedures for women seeking prophylaxis and treatment for pandemic influenza need to incorporate questions about the possibility of pregnancy. By including scenarios involving pregnant women in pandemic influenza preparedness exercises, public health professionals will have the opportunity to weigh the risks and benefits of anti-influenza medications in the context of a specific pandemic scenario. Their inclusion in preparedness exercises will help to identify gaps in our current capacity to provide optimal care for this high-risk population.

## Conclusions

Because of their risk for severe disease and death and the potential for risk for the fetus, pregnant women should be considered to be high-risk in the event of an influenza pandemic. Research into the effects of maternal influenza and its treatment on the pregnant woman and her fetus is sorely needed. Based on the limited information available, pregnant women who become ill with influenza should be treated aggressively with antifever therapy, and should adhere to standard recommendations for folic acid consumption (*9*). Given the limited data currently available, plans for prophylaxis and treatment for pandemic influenza will need to include reassessment of risks of influenza and risks and benefits of treatment strategies as a pandemic evolves. In addition to incorporating considerations specific to pregnant women into pandemic influenza planning efforts, strategies to communicate this guidance to pregnant women and their healthcare providers must be planned, developed, and tested. Only through consideration of all these issues, from research and planning to communications and intervention, will the health and well-being of pregnant women be ensured in a future influenza pandemic.
